# Atomic-scale imaging of CH_3_NH_3_PbI_3_ structure and its decomposition pathway

**DOI:** 10.1038/s41467-021-25832-9

**Published:** 2021-09-17

**Authors:** Shulin Chen, Changwei Wu, Bo Han, Zhetong Liu, Zhou Mi, Weizhong Hao, Jinjin Zhao, Xiao Wang, Qing Zhang, Kaihui Liu, Junlei Qi, Jian Cao, Jicai Feng, Dapeng Yu, Jiangyu Li, Peng Gao

**Affiliations:** 1 Electron Microscopy Laboratory, International Center for Quantum Materials, School of Physics, Peking University, Beijing, China; 2 State Key Laboratory of Advanced Welding and Joining, Harbin Institute of Technology, Harbin, China; 3 Shenzhen Key Laboratory of Nanobiomechanics, Shenzhen Institute of Advanced Technology, Chinese Academy of Sciences, Shenzhen, China; 4 School of Materials Science and Engineering, School of Mechanical Engineering, Shijiazhuang Tiedao University, Shijiazhuang, China; 5 Department of Materials Science and Engineering, Southern University of Science and Technology, Shenzhen, China; 6 Department of Materials Science and Engineering, College of Engineering, Peking University, Beijing, China; 7 State Key Laboratory for Mesoscopic Physics, School of Physics, Peking University, Beijing, China; 8 Collaborative Innovation Center of Quantum Matter, Beijing, China; 9 Department of Physics, South University of Science and Technology, Shenzhen, China; 10 Guangdong Provisional Key Laboratory of Functional Oxide Materials and Devices, Southern University of Science and Technology, Shenzhen, China; 11 Interdisciplinary Institute of Light-Element Quantum Materials and Research Center for Light-Element Advanced Materials, Peking University, Beijing, China

**Keywords:** Photovoltaics, Solar cells

## Abstract

Understanding the atomic structure and structural instability of organic-inorganic hybrid perovskites is the key to appreciate their remarkable photoelectric properties and understand failure mechanism. Here, using low-dose imaging technique by direct-detection electron-counting camera in a transmission electron microscope, we investigate the atomic structure and decomposition pathway of CH_3_NH_3_PbI_3_ (MAPbI_3_) at the atomic scale. We successfully image the atomic structure of perovskite in real space under ultra-low electron dose condition, and observe a two-step decomposition process, i.e., initial loss of MA^+^ followed by the collapse of perovskite structure into 6H-PbI_2_ with their critical threshold doses also determined. Interestingly, an intermediate phase (MA_0.5_PbI_3_) with locally ordered vacancies can robustly exist before perovskite collapses, enlightening strategies for prevention and recovery of perovskite structure during the degradation. Associated with the structure evolution, the bandgap gradually increases from ~1.6 eV to ~2.1 eV. In addition, it is found that C-N bonds can be readily destroyed under irradiation, releasing NH_3_ and HI and leaving hydrocarbons. These findings enhance our understanding of the photoelectric properties and failure mechanism of MAPbI_3_, providing potential strategies into material optimization.

## Abstract (web summary)

The knowledge of atomic structure and structural instability of hybrid perovskites is crucial to understand their photoelectric properties and failure mechanism. Here, the authors utilise low-dose TEM imaging technique to investigate the atomic structure and decomposition pathway of MAPbI_3_ at the atomic scale.

## Introduction

Organic-inorganic hybrid perovskites (OIHPs) have attracted great research interests as promising materials for the next generation photovoltaic energy harvesting^1,2^, electro-optic detection^3,4^ and all-optical conversion^5,6^. Their remarkable properties are underpinned by atomic structures of hybrid perovskites^7^, ABX_3_, with organic species such as CH_3_NH_3_^+^ (MA^+^) and CH(NH_2_)_2_^+^ (FA^+^) occupying A-site and inorganic Pb^2+^ in B-site surrounded by X-octahedron formed by halogen elements like I^−^ and Br^−^. In particular, the corner-sharing [PbI_6_]^4−^ octahedron is believed to be beneficial for carrier diffusion^8,9^, while its distortion under chemical strain^10^ makes the band gap tunable, ideal for photovoltaic conversion. Moreover, the organic cations as well as the hydrogen bonding may lead to spontaneous polarization and ferroelectricity^11^, which promotes the separation of photoexcited electron-hole pairs, and thus reduces the recombination and improves the carrier lifetime^12^. These characteristics are responsible for the promising optoelectronic properties including high carrier mobility, long charge diffusion length and superior power conversion efficiency^13^. Nevertheless, the exact atomic structure of OIHPs is still unsettled, with two possible space groups, polar I4cm and nonpolar I4/mcm still hotly debated depending on the orientations of polar ions such as MA^+^^ 14^. While many perovskite oxides are polar with strong ferroelectricity, the polarity of OIHPs has yet to be firmly established^15^.

The lack of detailed understanding on atomic structure of OIHPs is largely due to the incapability to image OIHPs at the atomic scale^16,17^. It is well known that OIHPs are quite unstable and prone to decomposition under electron beam irradiation^18–21^. While much progress has been made in transmission electron microscopy (TEM) characterizations of OIHPs, direct visualization of atomic structure remains to be elusive. Initial TEM studies at low doses are mainly observing the morphology evolutions^18^ and structure transitions by reciprocal-space electron diffraction (ED) techniques^19,20^, and many of the earlier studies mislabeled the decomposition product PbI_2_ as MAPbI_3_^22–24^. With the help of direct-detection electron-counting (DDEC) camera, high-resolution TEM (HRTEM) image of CH_3_NH_3_PbBr_3_ has been successfully obtained, which is much more stable than MAPbI_3_, though the observed off-centered MA^+^ with different orientations has not been well substantiated^16^. Recently, low-dose scanning transmission electron microscopy (STEM) technique provides atomic-scale insights into crystalline defects of CH(NH_2_)_2_PbI_3_ (FAPbI_3_), though the obtained atomic structures have already been damaged due to the large doses involved (53–221 e Å^−2^)^25^. Cryo-HRTEM has been used to image MAPbI_3_ at 100 e Å^−2^, yet the corresponding fast Fourier transform (FFT) pattern lacks (002) reflection, indicating substantial beam damage^26^. Furthermore, Li et al. found that superstructure reflections, a sign of structural transition due to beam damage, have already appeared at a dose as low as 7.6 e Å^−2^ (Ref. ^27^), and under Cryo-TEM, rapid amorphization has also been observed^18,28^. Indeed, the damage-free pristine structure of MAPbI_3_ has not been imaged at the atomic scale, and the corresponding real-space degradation pathway is yet to be established, thus motivating this study.

It is well known that STEM imaging introduces comparably larger dose and damage than low-dose HRTEM, while the contrast of HRTEM is sensitive to imaging condition, making it difficult to identify the specific atomic columns of MAPbI_3_^29^. To overcome these difficulties, we adopted DDEC camera combined with an imaging technique using a negative value of the spherical-aberration coefficient^30^, and we have successfully captured the atomic structure of MAPbI_3_ in real space at a dose as low as 0.7 e Å^−2^, ensuring minimum beam damage if any. We further observed a two-step degradation pathway at the atomic scale, initiated with the loss of MA^+^ to form a superstructure MA_0.5_PbI_3_ with ordered MA^+^ vacancies (${{{\mbox{V}}}}_{{{\mbox{MA}}}}^{-}$), followed by the diffusion of I^−^ and Pb^2+^ to form the decomposed 6H-PbI_2_, with the corresponding critical doses also identified. During the process, C–N bonds can be destroyed under irradiation, releasing NH_3_ and HI and leaving hydrocarbons. The continuous structure transformations result in gradually increased bandgap, which is confirmed by scanning electron microscope cathodoluminescence (SEM-CL) experiments and validated by density functional theory (DFT) calculations. The direct visualization of the structure and degradation process at the atomic scale provides valuable sights into understanding the properties and stability of OIHPs. Furthermore, the emergence of superstructure before the collapse of perovskite framework also points toward a strategy for stabilizing the materials during the degradation.

## Results

### Identification of damage-free threshold dose

**Fig. 1:**
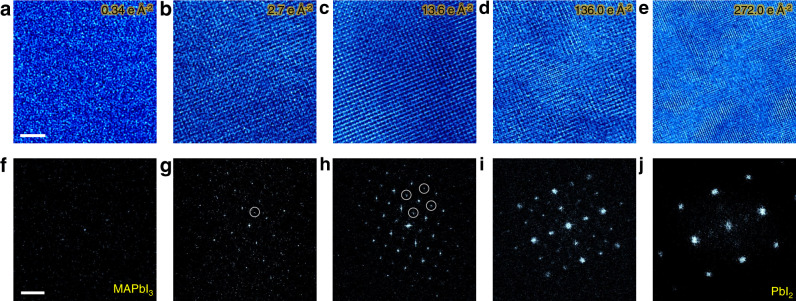
Tracking structure evolution during the decomposition of MAPbI_3_. **a**–**e** Time-series HRTEM images during the degradation of MAPbI_3_ under electron beam irradiation. The corresponding doses are marked on each panel. Scale bar, 4 nm. **f**–**j** The corresponding FFT patterns from MAPbI_3_ to 6H-PbI_2_. Circles indicate the superstructure diffraction reflections. Scale bar 2 nm^−1^.

MAPbI_3_ nanocrystals with 10–20 nm size and good crystalline (Supplementary Fig. 1) are chosen for low-dose imaging. Using DDEC camera, HRTEM images of MAPbI_3_ can be acquired at low doses as shown in Fig. 1. It is noted that sufficient dose (Supplementary Fig. 2) is needed to obtain images with good quality and superstructure diffraction reflections appear due to the generation of intermediate phases when the dose is larger than 2.7 e Å^−2^ (Fig. 1b, g). Judging from the corresponding FFT patterns, the [001] MAPbI_3_ with intermediate phases gradually transforms at 13.6 e Å^−2^, and finally decomposes into [$\bar{4}$41] or [48$\bar{1}$] 6H-PbI_2_ (Supplementary Fig. 3) at 272.0 e Å^−2^. Such a 6H-PbI_2_ product has also been observed during the degradation of polycrystal MAPbI_3_^21^. Thus the threshold dose for MAPbI_3_ without forming superstructures is determined to be 2.7 e Å^−2^ while 272.0 e Å^−2^ for the complete decomposition into PbI_2_. These determined doses can guide the future TEM characterizations of MAPbI_3_, especially for in situ ones such as under heat, electric field etc, for which the effect of electron beam irradiation is generally more significant due to the larger dose.

### Atomic-imaging of MAPbI_3_ structure and the intermediate phase

**Fig. 2:**
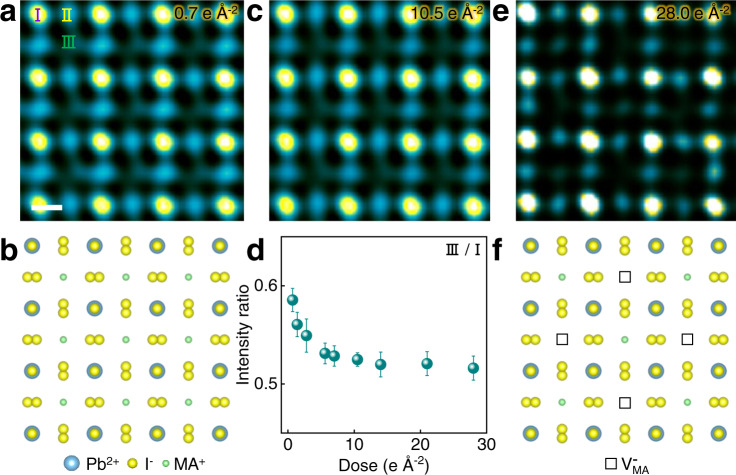
Atomic-imaging of the loss of MA^+^ and intermediate phase. **a** HRTEM image acquired at 0.7 e Å^−2^. ‘I’, ‘II’ and ‘III’ columns correspond to Pb^2+^-I^−^, I^−^, and MA^+^ columns respectively. Scale bar, 3 Å. **b** Structural model of tetragonal MAPbI_3_. **c** HRTEM image acquired at 10.5 e Å^−2^. **d** Intensity ratio of ‘III’ to ‘I’ column with increased doses. Nine data points of ‘III’ and ‘I’ type columns are used to obtain nine intensity ratios at each dose. The error bars represent standard deviations. **e** HRTEM image acquired at 28.0 e Å^−2^. **f** Structural model of MA_0.5_PbI_3_. The squares indicate ${{{\mbox{V}}}}_{{{\mbox{MA}}}}^{-}$.

We then investigate the atomic structure via an imaging technique using a negative value of the spherical-aberration coefficient (Cs), which has enabled the successful observation of both light and heavy elements in oxide perovskite^30^. Figure 2a is the HRTEM image acquired at a negative Cs with an overfocus, wherein the brightest ‘I’ column, second brightest ‘II’ column and the darkest ‘III’ column can be distinguished. By comparing the atomic structure features of MAPbI_3_ (Fig. 2b) and HRTEM simulations (Supplementary Fig. 4), ‘I’, ‘II’ and ‘III’ columns are identified to be Pb^2+^-I^−^, I^−^ and MA^+^, respectively. With increased dose, the intensity of MA^+^ is decreased at 10.5 e Å^−2^ as shown in Fig. 2c. The quantitative intensity analysis (Fig. 2d) further verifies that the intensity of MA^+^ progressively decreases within the initial 10.5 e Å^−2^, and then remains stable until 28.0 e Å^−2^ before subsequent gradual increase (Supplementary Fig. 5). The decreased intensity is caused by the formation of ${{{\mbox{V}}}}_{{{\mbox{MA}}}}^{-}$ ^31^ while the unchanged intensity is likely resulted from a relatively stable intermediate phase with the preserved perovskite framework. The subsequently increased intensity is resulted from the diffusion of I^−^ and Pb^2+^, as discussed in the following. Figure 2e and Supplementary Fig. 6 further show ${{{\mbox{V}}}}_{{{\mbox{MA}}}}^{-}$ appears at every other ‘III’ column, as illustrated in Fig. 2f. Such a cation-vacancy-ordered structure with superstructure reflections (Supplementary Fig. 7) corresponds to MA_0.5_PbI_3_, whose stability is verified by molecular dynamic simulation (Supplementary Fig. 8). Accordingly, it is concluded that the loss of MA^+^ starts even at 1.0 e Å^−2^ and reaches a balanced state between ~10.5 and 28.0 e Å^−2^ to form ordered ${{{\mbox{V}}}}_{{{\mbox{MA}}}}^{-}$, wherein the perovskite structure framework is preserved.

### Evolution of the electronic structure and chemical bonding

**Fig. 3:**
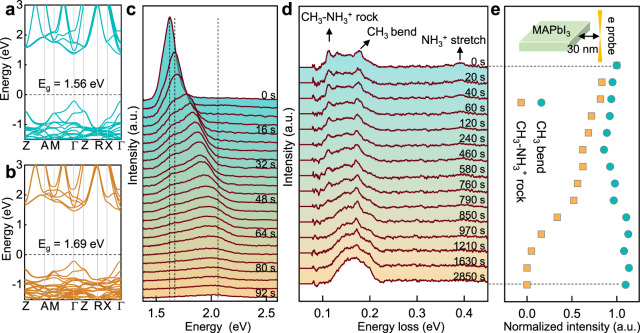
Electronic structure and chemical bonding evolutions during the degradation. **a, b** Electronic structures of MAPbI_3_ and MA_0.5_PbI_3_. The Fermi level is set to zero. **c** Time-series CL spectra showing the bandgap gradually increases from 1.6 eV to 2.05 eV. The dashed lines show the calculated bandgaps at 1.56, 1.69 and 2.15 eV for MAPbI_3_, MA_0.5_PbI_3_ and 6H-PbI_2_, respectively. **d** Time-series vibrational spectra under ‘aloof’ mode with the electron probe ~30 nm away from MAPbI_3_. Black arrows indicate the peaks related with CH_3_-NH_3_^+^ rock, CH_3_ bend and NH_3_^+^ stretch. The background was subtracted by the power-law function. **e** Evolutions of normalized intensities of CH_3_ bending (cyan circles) and CH_3_-NH_3_^+^ rock (orange squares) during the degradation. Inset is the schematic diagram of ‘aloof’ mode showing the electron probe is ~30 nm away from MAPbI_3_ for the vibrational spectroscopy measurements.

The effect of ordered ${{{\mbox{V}}}}_{{{\mbox{MA}}}}^{-}$ on its electronic structure is further investigated. Figure 3a, b show the calculated band structure of MAPbI_3_ and MA_0.5_PbI_3_. The band gap of MAPbI_3_ is 1.56 eV while it is 1.69 eV for MA_0.5_PbI_3_. The increased band gap is caused by the enhanced hybridization between I^−^-5p and Pb^2+^-6p atomic orbitals and the conduct band minimum shifting about 0.1 eV to the high energy level, as explained in Supplementary Fig. 9. To confirm this analysis, we also carried out SEM CL experiments. Supplementary Fig. 10 shows the initial CL emission with a single excitonic peak at the photon energy of ~1.58 eV. Time-series CL emissions in Fig. 3c show that the observed peaks gradually become broader and shift to higher energy (2.05 eV) with the excitonic peak intensity decreasing. Such blue-shift is associated with the electron-beam-induced phase transformations^32^, i.e., forming the intermediate phases and decomposing into 6H-PbI_2_, considering that the calculated bandgaps of MAPbI_3_, MA_0.5_PbI_3_ and 6H-PbI_2_ are 1.56, 1.69, and 2.15 eV (Supplementary Fig. 11), respectively, in good agreement with the experimental observation.

In addition to electronic structure evolutions, it is worth investigating how the chemical bonding within organic components evolves (Fig. 3d, e) during the degradation. Vibrational electron energy loss spectroscopy operated at ‘aloof’ mode^33^ (inset of Fig. 3e), which enables the control of damage by changing the distance between electron beam and sample^33^, is used to obtain the characteristic vibrational modes of MAPbI_3_ (Fig. 3d). We can observe the vibrational signals of CH_3_-NH_3_^+^ rock at 113 meV, CH_3_ bend at 177 meV, and NH_3_^+^ stretch at 391 meV^34^. Time-series vibrational spectra show that the peaks of CH_3_-NH_3_^+^ rock and NH_3_^+^ stretch gradually disappear with increased time, suggesting the breakage of chemical bonds and/or escape of the certain gas. The extracted intensities of C–N and C–H bonds are shown in Fig. 3e and the processing details are shown in Supplementary Fig. 12. It is observed that the intensity of C–H bonds does not decrease during the degradation, suggesting the negligible release of carbonaceous gas. Such C–H bend signals can come from (–CH_2_–CH_2_–)^35–37^ or CH_3_I, while previous energy-dispersive spectroscopy^19^ and X-ray photoelectron spectroscopy^38^ results show that the atomic ratio of I to Pb for the beam-induced decomposition product is ~2, suggesting it to be PbI_2_ instead of CH_3_I. Thus, the C–H bend signal in the final byproduct likely comes from (–CH_2_–CH_2_–)^35–37^. In addition, the peak of CH_3_-NH_3_^+^ rock gradually disappears, suggesting its breakage, which leads to the formation and escape of volatile NH_3_, further explaining the gradually decreased intensity of the N–H bond (Fig. 3d) and the drop of N content to 0 (Ref. ^38^). Such degradation process has also been proposed in the previous study based on the exposure to moisture^39^, indicating similarities in degradation mechanism between the electron beam irradiation and other stimuli.

### Atomic-scale observation of I^−^ and Pb^2+^ diffusion

**Fig. 4:**
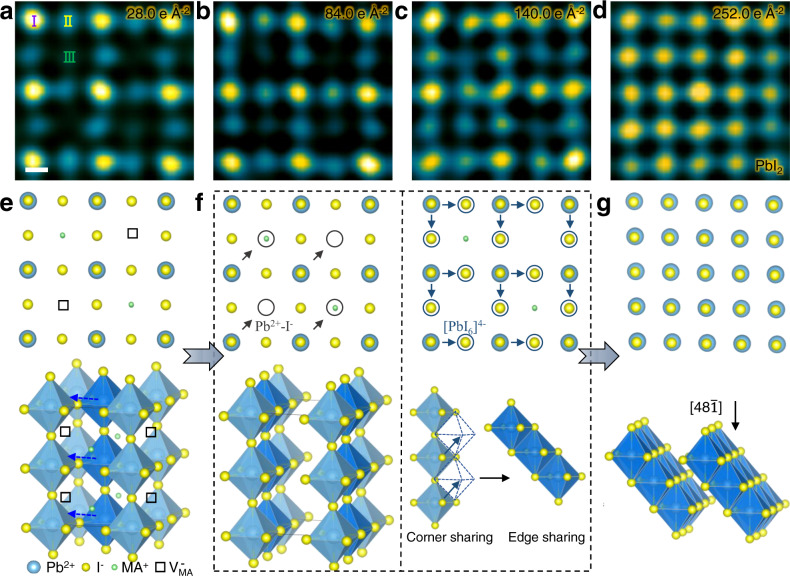
Atomic-scale imaging of the decomposition pathway. **a**–**d** HRTEM images with increased doses during the degradation into PbI_2_. The corresponding doses are marked on each panel. Scale bar, 2 Å. **e** Atomic structure of MA_0.5_PbI_3_. Black squares indicate ${{{\mbox{V}}}}_{{{\mbox{MA}}}}^{-}$. **f** Atomic structures to illustrate two kinds of I^−^ and Pb^2+^ diffusion. Left panel shows I^−^ and Pb^2+^ diffusing to ${{{\mbox{V}}}}_{{{\mbox{MA}}}}^{-}$ while the right panel illustrates the [PbI_6_]^4−^ octahedron slipping from corner sharing to edge sharing. **g** Atomic structure of PbI_2_.

Based on above study, we further investigate the atomic-scale decomposition pathway of MAPbI_3_. Figure 4a is the structure of perovskite with ${{{\mbox{V}}}}_{{{\mbox{MA}}}}^{-}$, as illustrated in Fig. 4e. With increased doses, it is observed that the intensities of these three columns gradually change (Fig. 4b–d). A quantitative analysis of the intensity changes (Supplementary Fig. 13) shows that the intensities of ‘I’ columns initially increase and then gradually decrease while the intensities of ‘II’ columns continuously increase and the intensities of ‘III’ columns initially decrease and then gradually increase. Finally, intensities of all three type columns converge, indicating the formation of PbI_2_. The initial intensity decrease of MA^+^ column results from the formation of ${{{\mbox{V}}}}_{{{\mbox{MA}}}}^{-}$, while the following increased intensity of MA^+^ column and decreased intensity of Pb^2+^-I^−^ column are believed to be caused by two kinds of I^−^ and Pb^2+^ diffusion as illustrated in Fig. 4f. One is the diffusion of I^−^ and Pb^2+^ into ${{{\mbox{V}}}}_{{{\mbox{MA}}}}^{-}$ (Supplementary Fig. 14) while the other is caused by the [PbI_6_]^4−^ octahedron slipping from corner sharing to edge sharing. Finally, the structure gradually evolves to PbI_2_. Figure 4e–g illustrate the atomic-scale structural evolution and the related ion migration during the decomposition from MAPbI_3_, MA_0.5_PbI_3_, to the final PbI_2_, mainly involving two processes, forming ${{{\mbox{V}}}}_{{{\mbox{MA}}}}^{-}\,$and the collapse of perovskite structure via the diffusion of Pb^2+^ and I^−^. While previous literature^21^ proposed a two-step transformation of polycrystalline MAPbI_3_: Pb-related defects form at grain boundary and then phase transforms into PbI_2_, here we have observed an intermediate phase with locally ordered MA^+^ vacancies and revealed the atomic-scale decomposition pathway from MAPbI_3_ into PbI_2_, which enhances our understanding on the OHIPs’ degradation.

## Discussion

The extreme beam sensitivity of OIHPs hinders atomic-resolution imaging and thus the detailed investigations on their structure-property relationships. By DDEC camera, we have determined that the threshold dose for superstructure formation is about 2.7 e Å^−2^, and perovskite collapses into PbI_2_ within 272.0 e Å^−2^, both of which are smaller than those measured by ED^19^. This is mainly because ED pattern is obtained from a comparably larger region and represents the average information. These threshold conditions can guide future TEM characterizations and encourage more atomic-scale investigations about OIHPs.

Atomic-scale imaging of MAPbI_3_ and its degradation pathway allows us to better understand properties of OIHPs. For example, the observed off-center displacements (up to ~30 pm) between different atom columns likely indicate the polar nature of this material^40^, although further studies are needed to fully clarify this point, including determining MA^+^ configuration^15^ and quantifying the effects from possible mistilt and residual aberrations^41^. Furthermore, from the energetic point of view, the electron beam can transfer energy to the materials, enable ions to overcome the diffusion barrier and thus induce structure transformations, which is similar to heat- or light-induced phase transformations and degradation. For example, the decomposition of MAPbI_3_ into PbI_2_ with the release of NH_3_ and HI^38^ has also been observed under light illumination^42^ and moisture atmosphere^39^. In addition, the increase of bandgap during the degradation has also been observed under illumination^43,44^. Therefore, our investigation of electron beam-induced decomposition pathway can also help understand how the devices fail in practical working conditions. Besides, the superstructure phase with additional reflections has been previously reported to be likely related with octahedra tilts^18^ or ordered iodine vacancies^19^ based on reciprocal-space ED analysis, our atomic-resolution imaging, however, has suggested that cation-ordered vacancies are more likely. Such an intermediate phase (MA_0.5_PbI_3_) with locally ordered vacancies can stably exist before perovskite collapses, suggesting the degraded structure with partial formation of ${{{\mbox{V}}}}_{{{\mbox{MA}}}}^{-}$ may be recovered. This likely sheds light into reversible photoinduced structural changes without forming PbI_2_^45^. Such self-healing behavior under illumination has also been observed in MAPbI_3_-based solar cells^46^. In addition, the loss of MA^+^ causes the increase of bandgap, which provides a potentially new strategy to tune the bandgap in constructing tandem solar cells^47^. Also, the increased bandgap facilitates multiwave electroluminescence emission, adjusting various color luminescence under increasing bias voltage^4,48^.

Ion migration in OIHP-based electronic device is regarded as one of the most important processes, which contributes to the phase segregation, hysteresis in J-V curves and device degradation^49^. Previous studies about ion migration are either based on calculations or macro-measurements^49^ like time of flight secondary ion mass spectroscopy^50^, conductive atomic force microscopy^51^, and energy-dispersive X-ray mappings^52,53^, all without achieving the atomic-scale resolution in real space. Our atomic-resolution imaging provides direct evidence for the diffusion of MA^+^, I^−^, and Pb^2+^ under electron beam irradiation, thus providing some insights into understanding ion-migration-induced phase transformations and degradation, and consequently the optimization of device performance. For example, since the gentle irradiation under illumination likely only causes the reversible formation of ${{{\mbox{V}}}}_{{{\mbox{MA}}}}^{-}$ with perovskite structure preserved, accordingly the device efficiency can be fully recovered at early degradation stages^54^. However, longer irradiation brings in I^−^ and Pb^2+^ diffusion to induce an irreversible transformation into PbI_2_, thus bringing in an irreversible device performance degradation. The irreversible performance decline has also been observed under elevated temperature^55,56^ and large bias^57^ due to the irreversible ion migration and structure degradation.

In summary, we have acquired the atomic structure of MAPbI_3_, determined the threshold doses during TEM characterizations, and clarified the atomic-scale ion migration during its degradation into PbI_2_. The degradation pathway is proposed to be a two-step, initialed by the loss of MA^+^ and followed by the diffusion of I^−^ and Pb^2+^ to form PbI_2_, during which C–N bonds can be destroyed under irradiation, releasing NH_3_ and HI and leaving hydrocarbons. Such degradation process leads to the gradual increase of bandgap. These findings can be used to guide the future TEM characterizations, enrich the understandings of the degradation mechanism and optimization strategies, and provide atomic-scale insights into understanding its fundamental properties.

## Methods

### MAPbI_3_ synthesis

MAPbI_3_ nanocrystals were bought from Xiamen Luman Technology Co., Ltd. Micro MAPbI_3_ was synthesized as previously reported^58^. Specifically, PbI_2_ and MAI were prepared in γ-butyrolactone (GBL) with molar ratio 1:1 and the concentration of 1.3 mol L^−1^. Then they were stirred at 70 °C for 12 h to obtain the precursor solution. After, the  precursor solution was filtered using polytetrafluoroethylene (PTFE) filter with 0.22 μm pore size. Two pieces of fluorine-doped tin oxide (FTO)/TiO_2_ substrates were clamped together and vertically and partially soaked in MAPbI_3_ precursor solution (10 ml) at 120 °C. Then the precursor solution was added twice one day in the nitrogen glove box. After several days, the substrates with single-crystal MAPbI_3_ film were brought out, and dried at 120 °C for 10 min in nitrogen.

### Characterization and image analysis

ED patterns and HRTEM images were acquired at an aberration corrected FEI electron microscope (Titan Cubed Themis G2) operated at 300 kV. The Cs value is ~6.8 μm. Before acquiring images, the illumination range was set to be ~3 μm in diameter. To shorten the exposure time to electron beam, we adjusted the defocus and the astigmatism well in one ~3-μm region, then blanked beam, and moved to another ~3-μm region to acquire the HRTEM image. HRTEM images were acquired at a magnification of 77,000 by DDEC camera in electron-counting mode with the dose fractionation function. The correction of drift was achieved by using the DigitalMicrograph software by cross-correlation. The original image contains 40 subframes in 4 s and every 2 subframes were summed to enhance the contrast for a more accurate estimation of drift. Hanning window and Bandpass filters were combined to improve the accuracy of the cross correlation. HRTEM images in Figs. 1, 2e, and 4, and Supplementary Figs. 2, 6, and 7 have been ABSF-filtered. HRTEM images in Fig. 2a, c have been first ABSF-filtered and then averaged from multiple regions using a home-made MATLAB code to reduce noise.

The morphology was characterized by SEM (FEI Quanta 200 F) and CL spectrum was acquired using Rainbow-CL of Beijing Goldenscope Technology Co., Ltd at 5 kV, spotsize 4. Each single CL spectrum was acquired using 4 s. Vibrational spectra characterizations were obtained under 30 kV using Nion U-HERMES200 electron microscope. Each spectrum was stacked from 200 single spectrum, obtained using 800 ms, and the processing details were shown in Supplementary Fig. 12. The simulated ED patterns were obtained by the SingleCrystal (Crystalmaker) software. Structural models were acquired using Vesta software.

### DFT calculation

Our first-principles calculations were performed within the framework of DFT as implemented in the Vienna ab initio simulation package^59,60^. The ion-electron interaction was depicted by projector augmented-wave method^61^. The electron exchange correlation was treated by the generalized gradient approximation with Perdew–Bruke–Ernzerhof functional^62^. A kinetic cutoff energy was set as 500 eV for the Kohn–Sham orbitals being expanded in the plane-wave basis. The atomic positions were fully optimized with a conjugate gradient algorithm until the Hellman–Feynman force on each atom are less than 0.01 eV/Å^63^. The Monkhorst-Pack k- point meshes was sampled as 9 × 9 × 7^64^.

### Ab initio molecular dynamics simulation

We performed the ab initio molecular dynamic (AIMD) simulation. The plane-wave cutoff was set as 500 eV and the Brillouin zone is sampled at the Γ point. The AIMD was performed in the canonical ensemble at 300 K.

## Supplementary information

Supplementary Information

## Data Availability

The authors declare that all relevant data are included in the paper and Supplementary Information files and are available from the corresponding author upon reasonable request.

## Code availability

Additional data including the codes are available from the corresponding author upon reasonable request.
