# Supplementary material for: Atomic-scale imaging of CH3NH3PbI3 structure and its decomposition pathway
Source: Nat Commun. 2021 Sep 17;12:5516. doi: 10.1038/s41467-021-25832-9 (PMC8448763; doi:10.1038/s41467-021-25832-9)
Supplement: Supplementary file 1 — Supplementary Information [file 41467_2021_25832_MOESM1_ESM.pdf]

Supplementary Information for

Atomic-scale imaging of $CH_3NH_3PbI_3$ structure and its decomposition pathway

Chen *et al.*

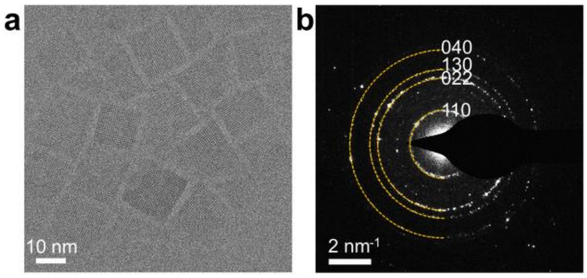

Supplementary Fig. 1 Morphology and crystalline of $MAPbI_3$. a HRTEM image of $MAPbI_3$ nano-crystals and **b** the corresponding electron diffraction pattern. Yellow half-circles indicate the corresponding planes of $MAPbI_3$.

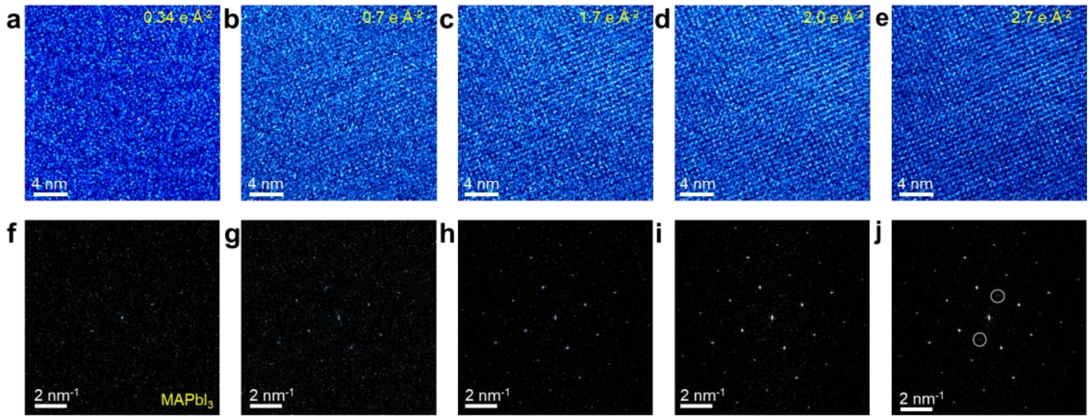

Supplementary Fig. 2 Low dose imaging of $MAPbI_3$ a-e Time-series HRTEM images of $MAPbI_3$ under electron beam irradiation. The corresponding doses are marked on each panel. **f-j** The corresponding FFT patterns. Circles indicate the superstructure reflections.

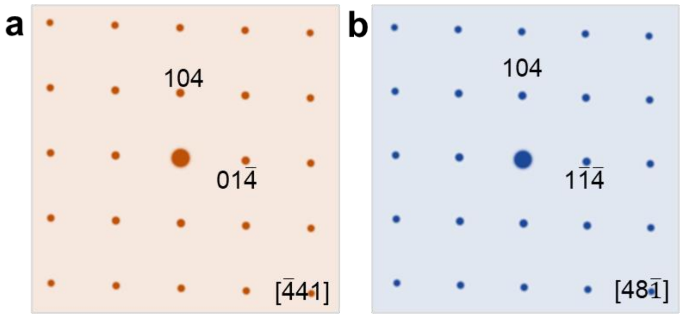

Supplementary Fig. 3 Simulated electron diffraction pattern of 6H-PbI_2 along **a** [$\bar{4}$41] and **b** [48$\bar{1}$] direction.

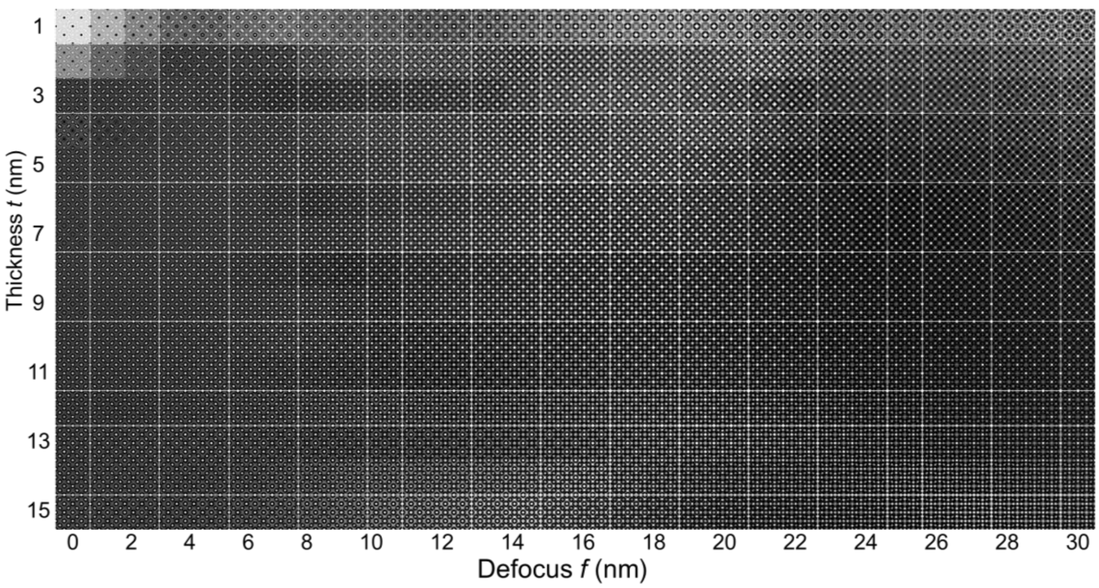

Supplementary Fig. 4 HRTEM simulation of $MAPbI_3$ under different thickness and defocus.

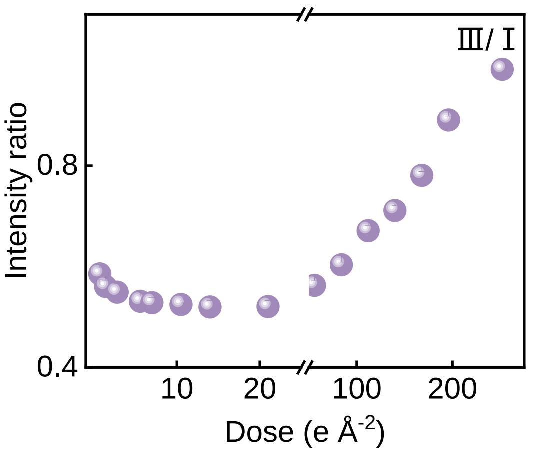

Supplementary Fig. 5 Intensity ratio of 'III' to 'I' atomic column with increased dose.

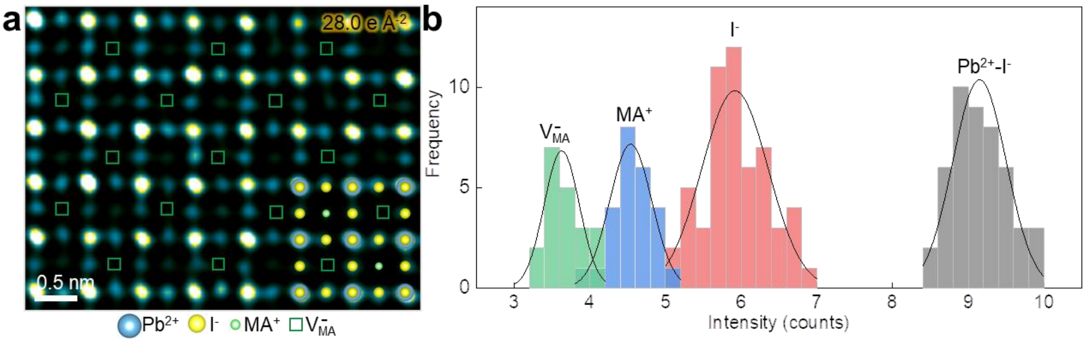

Supplementary Fig. 6 Atomic structure of $MA_{0.5}PbI_3$. a HRTEM images at 28.0 e $Å^{-2}$. The squares indicate ordered MA^+ vacancies (V^-_{MA}). **b** Intensities distribution of each atomic columns showing four types of columns including Pb^{2+}-I^-, I^-, MA^+ and V^-_{MA}.

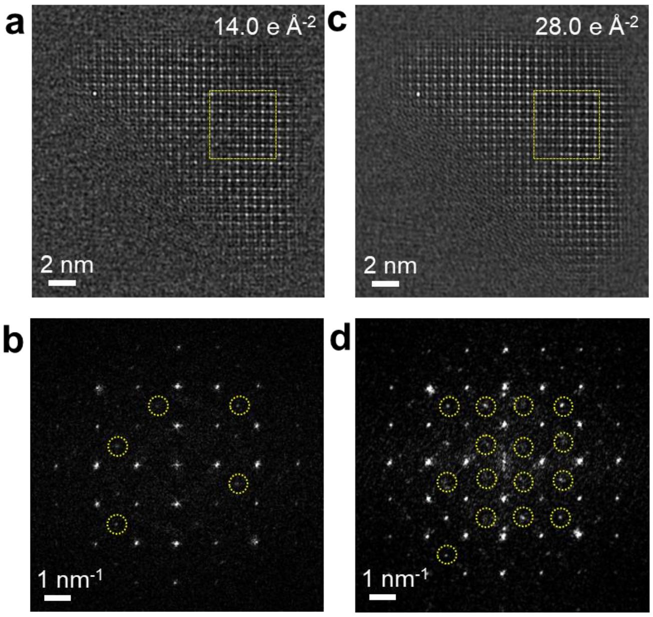

Supplementary Fig. 7 Low dose imaging $MAPbI_3$ and intermediate phase. a, c HRTEM images at 14.0 and 28.0 e Å^{-2}. **b, d** The corresponding FFT patterns from the yellow squares in **a** and **c**. Yellow circles indicate the additional superstructure reflections.

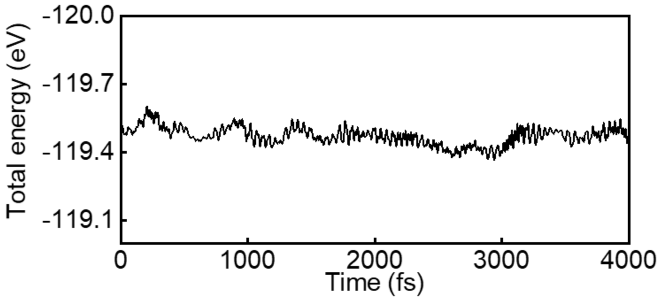

Supplementary Fig. 8 Molecular dynamic simulation of $MA_{0.5}PbI_3$. The corresponding energy is convergent, suggesting the structure of $MA_{0.5}PbI_3$ is stable.

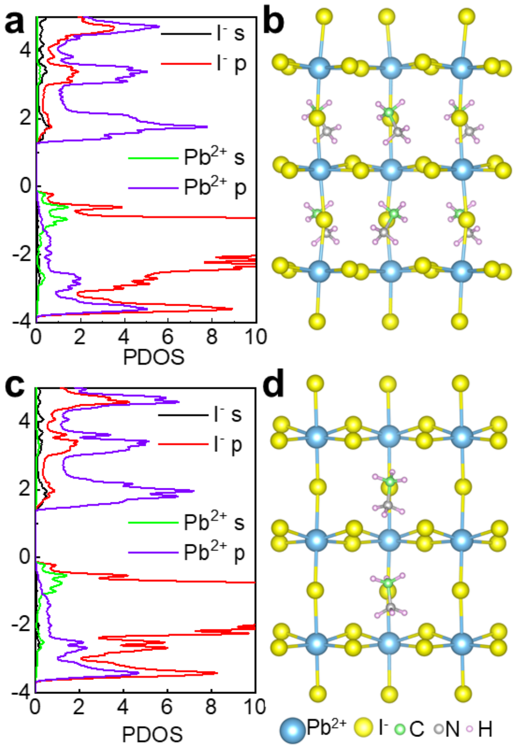

Supplementary Fig. 9 The projected density of states (PDOS) of $MAPbI_3$ and $MA_{0.5}PbI_3$. **a**, **c** PDOS of $MAPbI_3$ and $MA_{0.5}PbI_3$. **b**, **d** Atomic structure of $MAPbI_3$ and $MA_{0.5}PbI_3$. The orbitals of I^--5s, I^--5p, Pb^{2+}-6s and Pb^{2+}-6p are denoted by black, red, green, and blue solid lines, respectively. The valence bands of $MAPbI_3$ close to Fermi level are mainly derived from I^--5p orbitals and Pb^{2+}-6s orbitals while the conduction bands near fermi level are mainly formed from Pb^{2+}-6p and I^--5p orbitals. In contrast, DOS of $MA_{0.5}PbI_3$ shows that the contribution of I^--5p orbital near conduct band minimum (CBM) is slightly larger than that of $MAPbI_3$, which enhances the hybridization between I^--5p and Pb^{2+}-6p and results in the CBM shifting to high energy level with about 0.1 eV.

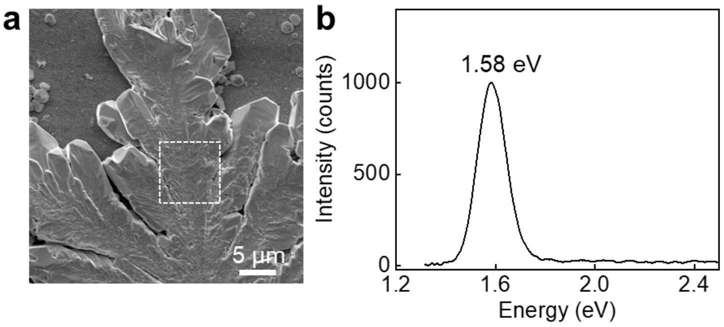

Supplementary Fig. 10 SEM and CL spectrum of $MAPbI_3$. a SEM image and **b** the corresponding CL spectrum obtained from the region marked by the dashed square in **a**.

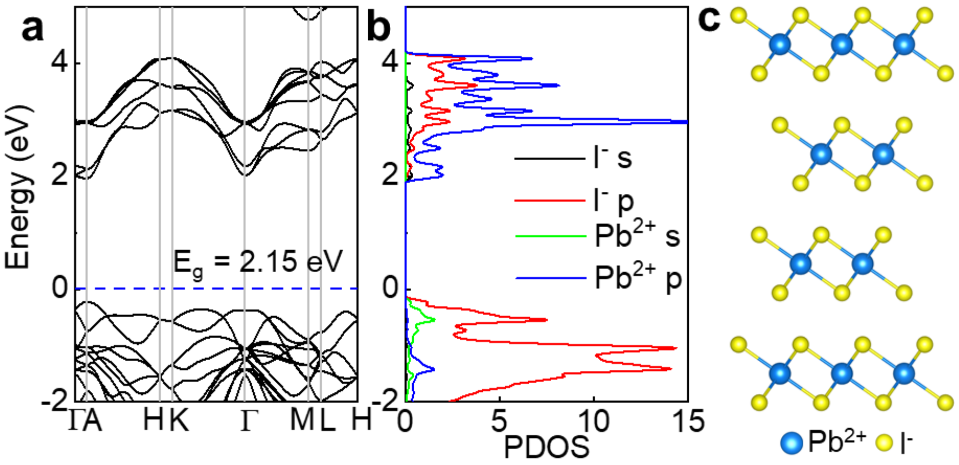

Supplementary Fig. 11 Structure of 6H-PbI$_2$. a Electronic structure, **b** PDOS and **c** atomic structure of 6H-PbI$_2$. The fermi level is set to zero. The I^--5s, I^--5p, Pb^{2+}-6s and Pb^{2+}-6p orbitals are denoted by black, red, green, and blue solid lines, respectively.

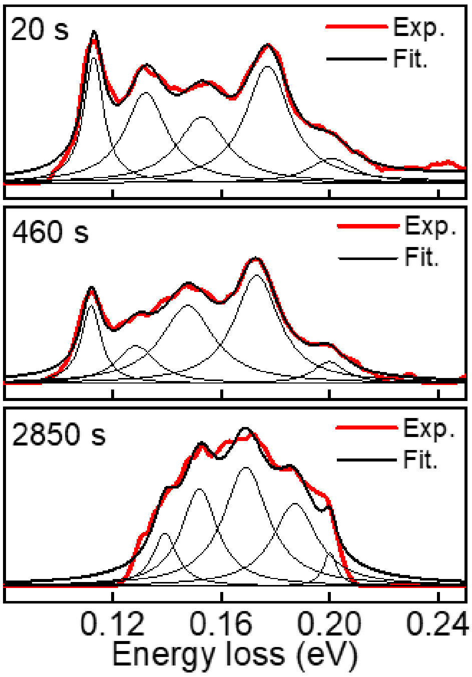

Supplementary Fig. 12 Fitting of vibrational spectra for $MAPbI_3$. The quantitative intensity of C-N and C-H are mainly based on the area between 107-117 meV and 170-180 meV. The peaks of final product can be fitted at 138, 150, 170, 190 and 200 meV. The peaks at 150 meV and 170 meV are likely from the damaged product –CH_2–CH_2–.

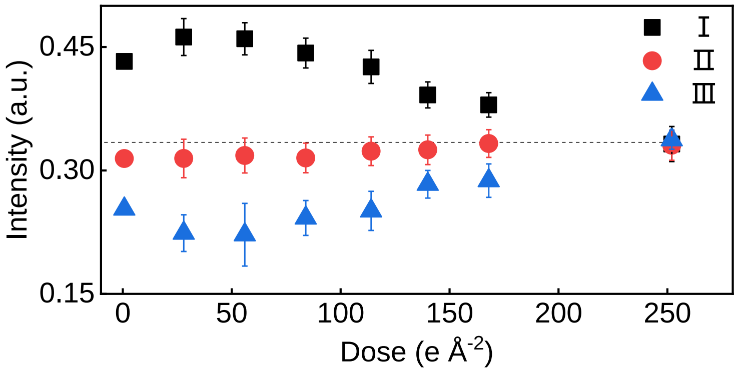

Supplementary Fig. 13 Normalized intensities of three types atomic columns are plotted with increased doses. Nine data points of 'I' type columns are averaged for each intensity of 'I' columns while ten data points of 'II' type columns and four data points of 'III' type columns are averaged for each intensity of 'II' and 'III' columns. The error bar represents the standard deviation. Normalized intensity is the ratio of each intensity to summed intensities of three types. The pristine intensity is obtained from the HRTEM image of $MAPbI_3$ at 0.7 e $Å^{-2}$ in Fig. 2a. The dashed line indicates the theoretical intensity ratio (0.33) of PbI_2 along the $[48\bar{1}]$ direction, wherein each column contains the same structure with alternating Pb^{2+} and I^-.

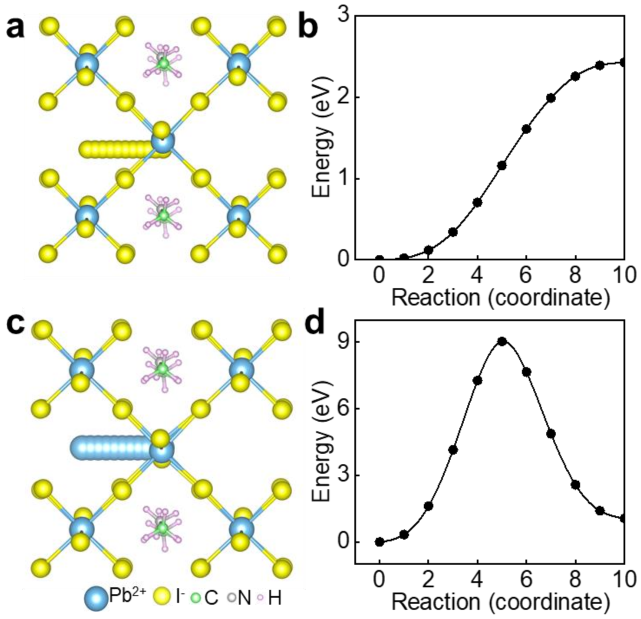

Supplementary Fig. 14 The diffusion of I$^-$ and Pb^{2+}. a, **c** Diffusion pathway of I$^-$ and Pb^{2+} into the V_{MA}^- in $MA_{0.5}PbI_3$. **b**, **d** The corresponding energy profiles. The corresponding diffusion energy barrier for I$^-$ and Pb^{2+} is ~ 2.43 and 9.06 eV respectively.
